# What Makes a Good Plant Invader?

**DOI:** 10.3390/life13071596

**Published:** 2023-07-20

**Authors:** Helena Korpelainen, Maria Pietiläinen

**Affiliations:** Department of Agricultural Sciences, Viikki Plant Science Centre, P.O. Box 27, FI-00014 University of Helsinki, Finland; maria.pietilainen@helsinki.fi

**Keywords:** invasive plants, dispersal, colonization, genetic variation, adaptation

## Abstract

We explored traits that promote plant invasions. External factors affecting invasion success consist of various abiotic and biotic constraints. How well plants perform under those depends on multiple characteristics, such as life history traits, genetic variation patterns, competitive and dispersal abilities, phenotypic plasticity, resistance, tolerance, and possibly allelopathic interactions. Since the introduction of invasive species is often connected with humans, their geographical distribution and differentiation may not reflect adaptation. However, a lack of adaptation may be compensated for by repeated introductions via mixing genotypes from multiple populations or through novel mutations. As a case study, we used data from the Global Invasive Species Database of IUCN and attempted to reveal factors contributing to invasiveness. The most prevalent features are that the dispersal is strongly human assisted, many species are used as ornamentals, disturbed habitats are favored, and most species are perennial. Distribution features show that the worst invasive species typically have a narrower native distribution, but both groups, i.e., most serious invasive and other listed invasive species, have commonly developed a multicontinental distribution. The change in the multicontinental distribution from 6% to 63% in most serious invasive species reflects their effectiveness in global dispersal and establishment. High proportions of invasive species in both groups have mixed reproduction systems, i.e., they have the ability to propagate both sexually and asexually (57% and 50%, respectively). This provides flexibility for spreading and establishment. A lower proportion of the worst invasive species was mainly/only sexual (23%, often hermaphrodites) when compared to other invasive plants (40%). In the case of sexual reproduction, hermaphroditism combined with self-compatibility may enhance invasiveness, since selfing allows fertilization and recombination even under low population densities. Overall, the ability for asexual propagation and, in the case of sexuality, hermaphroditism, is an asset in the invasion process.

## 1. Introduction

Non-indigenous species are species distributed outside their historic and native range. It has been proposed that a non-indigenous species must go through three stages to become invasive [1]. Firstly, individuals of the species must disperse from their native range to a new area. Secondly, after the introduction, the individuals must survive and reproduce in the new area and become established. Thirdly, once established, the non-indigenous species will increase in number, expand its geographic range, and become a threat to the ecosystem, i.e., become invasive. Thus, the success of invasion depends on the combination of dispersal and demography in a non-native region, and these are affected by the processes of post-dispersal adaptation, genetic diversity, and phenotypic plasticity. However, these processes are not well understood [2,3,4]. However, it is well known that invasive species threaten native biodiversity. Based on evidence from metacommunity models, it has been shown that species introductions could disrupt species coexistence, generating extinction debts, especially when combined with other forms of anthropogenic environmental changes [5]. Therefore, the control and eradication of invasive species are essential for the conservation of native species, biodiversity, and ecosystem function, e.g., plant–pollinator networks [6].

The introduction of alien species to new regions, either intentionally or accidentally, is often presumed to be connected with human activities, such as agriculture, horticulture, aquaculture, trade, transportation, and recreation, and is often accidental [2,4,7]. Thus, alien species are unique compared to other species, as their geographical differentiation may not be a result of adaptive processes in response to environmental gradients and biotic interactions, because humans disrupt those natural processes that normally determine genetic diversity. In addition, invasive species often cause severe environmental impacts and may have large economic and social consequences [1]. Overall, compared with the natural range expansion, humans have massively increased the rate at which certain species colonize new areas.

The present paper compiles information on specific plant traits and environmental conditions that promote plant invasions and complicate the control of invasive plants in ecosystems. As a case study, we used data available in the Global Invasive Species Database of the International Union for Conservation of Nature (IUCN). In addition to the IUCN database, there are other databases of invasive plants, e.g., the European DAISIE database, with a broad taxonomic spectrum (no longer maintained), the North European and Baltic Network on Invasive Alien Species (NOBANIS, www.nobanis.org, accessed on 15 July 2023), the Global Compendium of Weeds (www.hear.org/gcw, accessed on 15 July 2023), with global coverage, and the more comprehensive Global Naturalized Alien Flora database (GloNAF, https://glonaf.org, accessed on 15 July 2023). Based on GloNAF, patterns in the diversity and geographic distribution of all naturalized and invasive plant species, the relationships between the numbers of naturalized, invasive and native species, etc., have been summarized, showing, for example, a strong correlation between the numbers of invasive and all naturalized taxa, which demonstrates that regions considered as invasion hotspots correspond to regions known to have the greatest frequency of naturalized species [8]. We recognize that the IUCN database used is not comprehensive and is evidently biased towards more widely investigated and internationally reported geographic regions. However, it provides suitable global information on invasive plant species for the present case study. By comparing data on the most serious and other listed invasive plant species, we attempted to improve the understanding of invasive plant species, and address a knowledge gap of factors that are important for a plant’s success as an invader.

## 2. Traits Important for Successful Plant Invasion

**Figure 1:**
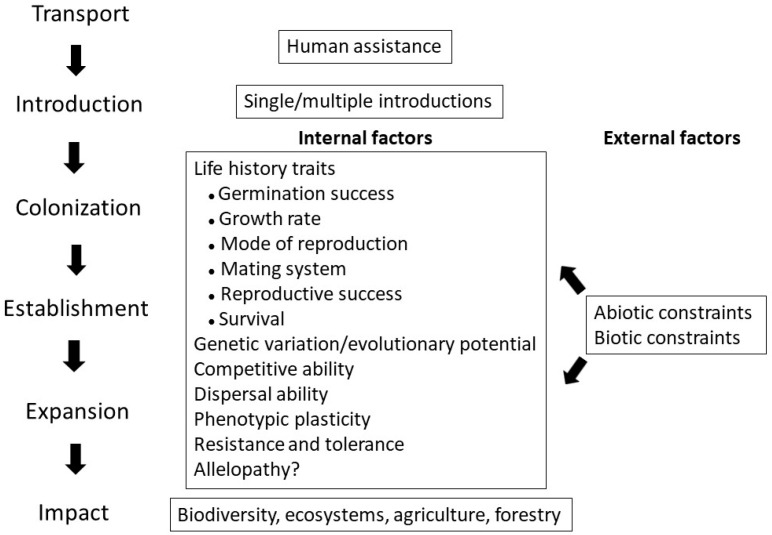
Internal and external factors affect the process of plant invasion from colonization to expansion, which may lead to impacts on biodiversity, ecosystems, forestry, and agricultural production. External factors consist of a range of abiotic and biotic constraints. The role of allelopathy is debated. The figure was prepared by the authors.

There has been great interest in determining mechanisms that make the switch from non-indigenous to invasive species happen and in revealing characteristics that contribute to a species’ invasive ability [9,10,11]. Figure 1 summarizes the process of plant invasion and shows the internal and external factors that affect the process. External factors consist of a range of abiotic and biotic constraints. How well a plant species performs under different conditions depends on a wide range of internal, partly overlapping characteristics. These include multiple life history traits, genetic variation patterns, competitive and dispersal abilities, phenotypic plasticity, resistance, tolerance, and possibly the effect of allelopathic interactions. Control and eradication measures of invasive plants become increasingly difficult as the invasion process progresses. Failure in those actions may result in harmful impacts on the biodiversity and ecosystem functioning, as well as on forestry and agricultural production.

One of the questions is whether the success of invaders is driven by post-introduction evolution or high plasticity. The idea that high phenotypic plasticity has contributed to the success of invasive species was proposed in the 1960s [12]. Indeed, it appears that many invasive plant species are more plastic in several traits than co-occurring non-invasive ones, but this plasticity is only sometimes associated with a fitness benefit based on a large-scale meta-analysis [10]. However, there are reports providing evidence of the importance of phenotypic plasticity in, e.g., morphological traits [13], reproductive traits [14], resource allocation [15], tolerance against herbivores [16], light use [17], and response to altered N supply [18]. However, investigations on *Plantago lanceolata* have indicated that phenotypic plasticity may have initially mediated its adaptation to stressful habitats, while the loss of plasticity associated with secondary invasion may inhibit its further range expansion [19]. Thus, although there is plenty of evidence for the role of phenotypic plasticity in promoting plant invasion, the evidence is not conclusive.

It has become evident that evolutionary phenomena connected with range expansion drive rapid life-history evolution [20,21]. The range expansion itself may be a process that selects for traits that aid invasion success at the range edge, such as enhanced dispersal and colonization, the latter including reproduction success and competitive ability [11,22,23]. In addition, invasive plants with high dispersal capabilities may benefit from increasing habitat fragmentation typically caused by humans. When comparing the effects of different types of life-history traits, it has been suggested that factors associated with evolutionary adaptation and population expansion might determine invasion success and extent, while traits related to the relative competitive ability of invasive species would determine the severity of impacts [24]. This has implications for the measures used to control invasive plants. 

In an investigation on life-history evolution in the invasive honeysuckle *Lonicera japonica*, it was discovered that its evolution within the area of invasion has resulted in an enhanced survival and increased growth rate that may drive spread, thus increasing the likelihood of further invasion [23]. In wind-dispersed plants, a trade-off is expected between dispersal and colonization ability, including germination success and growth that is mediated by seed size; smaller seeds tend to have a better dispersal ability, but possibly a poorer colonization ability [22]. However, the dispersal ability of the invasive wind-dispersed plant *Gladiolus queinzii* Kunze improves towards the range edges in eastern Australia, and this is mediated by a decreased seed size and faster germination time [11]. Thus, a change towards superior dispersal is not necessarily associated with an inferior colonization ability, even in wind-dispersed plants. 

The results on the salt marsh grass *Spartina alterniflora* (Loisel.) P.M.Peterson & Saarela support the hypothesis of the evolution of increased competitive ability, in this case through greater aboveground and belowground biomass [25]. In fact, belowground competition events may turn out to be important for the success of invaders. In experiments in the invasive grass species *Agrostis capillaris* L., it was shown that belowground competition can be more important in driving invasive plant impacts than aboveground competition in both low- and high-fertility ecosystems, including those experiencing N enrichment due to global change [26]. It was found that competition with the invader had large negative impacts on native species’ growth (biomass decreased by half), resource capture (total N content decreased by up to 75%) and even nutrient stoichiometry (tissue C:N ratios of native species increased).

## 3. Interactions between Genetic Traits and Environmental Conditions Promoting Plant Invasion

The suitability of the environment in the region of introduction is crucial for the establishment and spread of introduced species [1,27,28], as is their genetic composition and adaptability [4,29]. Mechanisms involving interactions between genetic traits and environmental conditions that lead to the adaptation of invasive species have been proposed. For instance, inbreeding × environment (I × E) interactions may have a role in invasion success. It has been suggested that a temporary or permanent release from stress in invaded habitats may alleviate the negative effects of insufficient genetic variation on fitness via I × E interactions and that may even generate adaptive genetic changes despite limited genetic variation [30]. 

There is evidence that populations spreading through favorable habitats can rapidly evolve high dispersal and reproductive rates, specifically at the expansion front, which further accelerates the spread velocity [28]. However, spreading populations are likely to encounter stressful conditions somewhere within the expansion range. How evolution during spread in favorable environments affects populations in harsher environments is largely unknown. When comparing evolutionary changes in performance under harsh conditions, i.e., under drought, interspecific competition, and heat stress, in *Arabidopsis thaliana* (L.) Heynh. populations, it was discovered that evolution during spread in favorable conditions may either constrain or extend the eventual range limit of species invasion [28]. A global demographic survey conducted for over 500 plant species showed that populations of invasive plants have superior potential to recover from disturbance compared to non-invasive ones [31]. Recovery from demographic disturbance was considered to be a measure of transient population amplification, linked to high levels of reproduction. 

Global change may reduce evolutionary advantages that native species have developed via adaptation. An investigation into the joint demographic effects of reproductive phenology and warming on the globally invasive thistle species *Carduus nutans* L. revealed a substantial shift toward the completion of the life cycle at younger ages [32]. Demographic modelling projected a 15% increase in this invader’s population growth rate. It was shown that rising temperatures accelerate its population growth by increasing the average size of reproducing individuals, the proportion of individuals that survive to reproduce, and the fraction that reproduce as annuals [32]. Increased growth and physiological performance under experimental conditions has been observed in the ground-creeping plant *Carpobrotus edulis* (L.) N.E. Br, which suggests that climate change would further promote its invasion [33]. *Parthenium hysterophorus* L. is a globally invasive weed with significant negative impacts. An analysis on *P. hysterophorus* showed that elevated atmospheric carbon dioxide concentration, high ambient temperatures, heatwaves, and droughts will facilitate its establishment and range expansion [34]. It is evident that climate change could aggravate problems caused by invasive plants and impose increasing management challenges. However, these results are not definite. For instance, a recent meta-analysis found that global change may rather offset than intensify the impacts of biological invasions [35].

## 4. Allelopathy and Herbivory Affecting Invasion Success

The allelopathy of plants, i.e., the production of biochemicals that influence the growth, survival, development, and reproduction of other organisms, arises through coevolution [36]. These compounds may impact plants, animals, and microbes. Plant-interkingdom interactions, especially those with soil-dwelling organisms, have been shown to be important factors in plant health and fitness, possibly providing a competitive advantage [37]. Allelopathy has been reported to influence the invasiveness of many species, for instance in the common lantana, *Lantana camara* L., a species native to tropical America [38], in the small mimosoid tree, *Leucaena leucocephala* (Lam.) de Wit, native to southern Mexico and Central America [39], and in the Mexican sunflower, *Tithonia diversifolia* (Hemsl.) A.Gray, native to Mexico and Central America [40]. A review of allelopathic information on 524 invasive plant species demonstrated that most of them produce allelochemicals with the potential to negatively affect the performance of native plants [40]. The released allelochemicals may suppress the regeneration process of other plant species by decreasing their germination and seedling growth and increasing their mortality. Therefore, allelopathy may support the invasive potential of *L. camara*, *T. diversifolia*, and others, and the formation of dense monospecies stands.

Herbivory can influence the evolution of plants’ chemical traits. Populations that escape selection from their ancestral herbivores may shift resources from defense to competitiveness [41,42]. Experiments on the goldenrod *Solidago altissima* L. have suggested that a release from herbivory is an important driver of plant adaptation in the invasive range [43]. However, studies on native and invasive populations of the African daisy, *Senecio pterophorus* DC., concluded that herbivory was not a selective factor after invasion [44]. Instead, the climate was a driver for post-invasion evolution, as suggested by plant adaptations to a drought cline in the native range, the analogous change in plant traits in invaded regions, and the convergence of vegetative traits between non-native and native plants under similar drought conditions. 

The Shifting Defense hypothesis gives a somewhat different view to the herbivory question among invasive plants [43,45,46]. Based on this hypothesis, invasive plants are likely to be released from specialist herbivores and, at the same time, they will encounter biotic resistance from local generalist herbivores in their new ranges. This will result in the evolution of a decreased defense against specialist herbivores and an increased defense against generalist herbivores. The meta-analysis conducted by Zhang et al. [46] indicated that two major mechanisms, i.e., release from specialist enemies and biotic resistance by generalist herbivores, may be pivotal in the evolution of defenses against herbivores. Thus, the analysis provided support for the Shifting Defense hypothesis. Additionally, seed predation can structure plant communities if there is selective foraging. It has been shown, for instance, that rodents prefer to eat the seeds of native grass species instead of the invasive cheatgrass, *Bromus tectorum* L. [47]. Rodent foraging reduced the establishment of each native species by at least 80%, but had no effect on the establishment of cheatgrass.

## 5. Pathways to Successful Plant Invasion

The genetic traits of introduced populations affect their capacity to expand in non-native regions. At introduction, they typically contain just a portion of the species’ gene pool and experience founder effects [29]. Such bottlenecks could inhibit the population growth and further expansion, as decreased genetic diversity is likely to result in inbreeding depression and reduced evolutionary potential, thus lowering the ability to respond to novel selection pressures. On the other hand, these populations may benefit from being freed from the biotic pressures of the former habitat. Nevertheless, many invasive species manage to adapt and occupy wider regions in new environments [21,29,48]. 

The genetic diversity of invasive populations can increase through multiple introductions and/or sexual reproduction along with recombination, especially if genetic variability is combined with phenotypic plasticity, facilitating adaptation and spread [49]. Clonal species may also gain significant amounts of genetic diversity via mutation [50]. Some invasive plants have even higher genetic diversity outside their native range [51]. The underlying mechanisms may include admixture (i.e., new genotypes arising from interbreeding among divergent source populations), hybridization, rapid mutation, and the expression of cryptic genetic variation, all potentially increasing the genetic diversity, colonization success, and adaptive potential of invasive species [4]. Moura et al. have suggested that the genetic variability provided by polyploidization has a positive impact on plant competitiveness, which may lead to an increased ability to colonize new environments [52]. In addition, it has been proposed that environmental stress may facilitate rapid adaptation through changes in the genome structure and function via interactions with transposable elements [53], and that environmental stress or other ‘genomic shock’ effects can lead to various patterns of gene expression re-programming and epigenetic changes that contribute to phenotypic variation [54]. These mechanisms can contribute to transgressive phenotypes and may explain the success of some plant invaders, especially in the case of low genetic variability.

To quantify the influence of demography and dispersal on the genetic diversity underlying adaptation, data covering 35 native and 18 non-native populations of the ribwort plantain *Plantago lanceolata* L. were used [4]. Simulations showed that dispersal would dilute the demographic influence on genetic diversity at local scales. However, non-native populations had a weaker spatial genetic structure that was not associated with environmental gradients but with a higher within-population genetic diversity. These findings showed that dispersal caused by multiple, long-distance, often human-mediated introductions has allowed invasive plant populations to overcome environmental constraints on genetic diversity, even without major demographic changes. Therefore, the impact of invasive plants may increase because of repeated introductions via mixing genotypes from multiple populations, providing sufficient adaptation.

Invasive populations becoming established in new regions can be a source of additional introductions, leading to a self-accelerating process. This phenomenon is called a bridgehead effect, where a successful primary population with high invasiveness is the source of multiple secondary introductions [55]. If this proposed pattern is correct, then global increases in future invasion rates would be expected. However, there is no conclusive evidence proving that the success of bridgehead populations always stems from the evolution of enhanced invasiveness [3]. Instead, an increased abundance of invasive individuals in the bridgehead region or the human transport networks may best explain the high frequency of secondary introductions [3]. In any case, independent of the underlying process generating secondary introductions, bridgehead populations are likely a source of new introductions.

## 6. A Case Study Comparing the Dispersal and Life Histories of Invasive Plants Varying in Their Prevalence

To investigate mechanisms and biological determinants that lead to plant invasions, we used information available on species in the Global Invasive Species Database of the International Union for Conservation of Nature (IUCN), the Species Survival Commission (SSC), and the Invasive Species Specialist Group (ISSG, http://www.iucngisd.org) as a source to obtain a list and basic background information for those plant species considered invasive. All plant species included in the above database were used in our analysis. The complete list covered 467 species (Table S1), which included 12 algae, 10 pteridophytes, and 445 seed plants (3 gymnosperms, 442 angiosperms). They were from 127 plant families (9 algal, 8 pteridophyte, 1 gymnosperm, and 109 angiosperm families). The three dominating families among these invasive species were Poaceae (12.0%), Fabaceae (10.5%), and Asteraceae (10.5%), followed by Rosaceae (3.6%) and Solanaceae (2.1%) and others with low frequencies. However, when reporting the prevalence of invasive species per family, it is important to consider the size of the family in question. Therefore, for the above five families, we calculated the ratios of the number of invasive species listed here and the estimated total number of species (total species number data from [56,57,58,59,60]). The proportions as percentages were as follows: 0.49% (56/11,500), 0.25% (49/19,500), 0.20% (49/25,000), 0.57% (17/3000), and 0.57% (17/3000) for Poaceae, Fabaceae, Asteraceae, Rosaceae, and Solanaceae, respectively. Based on these estimates, no conclusions can be made regarding potential differences in the tendency towards invasiveness between plant families. In addition, we compiled information of those plant species that belong to the ‘100 of the World’s Worst Invasive Alien Species’ list from IUCN. The plants in the list of worst invasive (WI) species included 35 species (2 algae, 1 pteridophyte, 1 gymnosperm and 31 angiosperms), Fabaceae being the most frequent family (16.2%). The rest (432 species) were considered to be other invasive species (OI).

Table 1 summarizes the main findings of the analysis among the worst and other invasive species, while more detailed data are available in Table S1. The taxonomy used in Table S1 is the same as in the Global Invasive Species Database of IUCN. The most prevalent features are that the dispersal in both WI and OI categories is strongly human assisted, with 30 (86%) and 392 (91%) species, respectively; many are ornamentals, 21 (60%) and 229 (53%) species, respectively; the species typically occur in disturbed habitats, 30 (94%) and 325 (80%) species, respectively; and most are perennial, 35 (100%) and 388 (90%) species, respectively. Distribution features show that WI typically have a narrower native distribution than OI, but species of both types have a multicontinental present distribution almost as frequently, i.e., in 22 (63%) and 253 (59%) species, respectively. The change in multicontinental distribution from 6% to 63% in WI reflects their effectiveness in global dispersal and establishment.

High proportions of both WI and OI had a mixed reproduction system, i.e., both sexual and asexual modes of reproduction, equaling 20 (57%) and 217 (50%) species, respectively (Table 1). This brings flexibility to spreading and establishment. A lower proportion of WI species was mainly/only sexual (8 species = 23%) when compared to OI plants (171 species = 40%) (χ^2^ test, *p* = 0.0503, non-significant but highly suggestive). In addition, hermaphrodites were more common among WIs (25 species = 80%) than in OIs (263 species = 64%) (χ^2^ test, *p* = 0.0697, non-significant but highly suggestive) when angiosperm plants were compared. In particular, hermaphroditism combined with self-compatibility may be a factor that enhances invasiveness because selfing guarantees fertilization and recombination even under low population densities. Interestingly, the obtained *p* values were close to statistical significance (notice the smallish sample size for WI species). Thus, the results suggest that when compared to OI species, WI species have a greater tendency to not rely on sexual reproduction, and when reproducing sexually having a stronger tendency towards hermaphroditism. Previously, it has been suggested that asexual reproduction prevails in many invasive plant species [61]. Clonality can be a special advantage in a novel environment, as one sex or individual plant can start to reproduce asexually and spread immediately. There are examples, e.g., the aquatic plants *Egeria densa* Planch. 1849, *Lagarosiphon major* Ridl. Moss ex Wager and *Hydrilla verticillate* (L.f.) Royle from the family Hydrocharitaceae, where only one sex has been introduced, yet the species has managed to spread successfully [62]. In comparison, the invasive terrestrial plants *Hieracium aurantiacum* L. (Asteraceae) and *Carpobrotus edulis* (L.) N.E.Br. (Aizoaceae) possess effective vegetative propagation, and individual clones can occupy very large regions [63,64]. Although there are many internal and external factors that influence the invasion success, an ability for asexual propagation and, in the case of sexuality, hermaphroditism are assets in the invasion process.

## 7. Conclusions

It is believed that invasive plant species are typically effective dispersers, can extend their range quickly, are successful at colonization and reproduction, and may tolerate a range of environmental conditions. In addition, invasive populations becoming established in new regions can be a source of additional introductions, leading to a self-accelerating process, called a bridgehead effect, where a successful primary population is the source of multiple secondary introductions. Due to commonly assisted introductions by humans or other vectors, invasive species are unique, as their geographical distribution and differentiation may not be as much a result of adaptive processes in response to environmental gradients and biotic interactions as in other species. Repeated introductions via mixing genotypes from multiple populations may improve the adaptation and impact of invasive plants. Furthermore, global change may reduce evolutionary advantages that native species have developed via adaptation, which could benefit invasive species and lead to greater harmful impacts on biodiversity and ecosystem functioning, as well as on the forestry and agricultural production. Control and eradication measures of invasive plants become increasingly difficult as the invasion process progresses.

There has been great interest in determining the mechanisms that make the switch from non-indigenous to invasive species happen and in revealing characteristics that contribute to a species’ invasive ability. However, it has become clear that the processes of plant invasion vary, and there is no single universal mechanism. For instance, different mechanisms involving interactions between genetic traits and environmental conditions that lead to the adaptation of invasive species have been proposed, e.g., inbreeding × environment (I × E) interactions may have a role in invasion success. Various inherent and external factors are recognized that affect the invasion process. External factors consist of a range of abiotic and biotic constraints. How well a plant species performs under those depends on a wide range of inherent, partly overlapping characteristics, such as multiple life history traits, the pattern of genetic variation, competitive and dispersal abilities, phenotypic plasticity, resistance and tolerance, herbivory, and allelopathic interactions.

As a case study, we used data available in the Global Invasive Species Database of the International Union for Conservation of Nature (IUCN). By compiling and comparing data on the worst (WI) and other listed invasive (OI) plant species, we attempted to improve the understanding of plant invasion. The three dominating families among these invasive species were Poaceae (12.0%), Fabaceae (10.5%), and Asteraceae (10.3%), followed by Rosaceae (3.6%) and Solanaceae (2.1%) and others with low frequencies. The most prevalent features were that the dispersal in both WI and OI is strongly human assisted, 86% and 91%, respectively; many are ornamentals, 60% and 53%, respectively; the species commonly occur in disturbed habitats, 94% and 80%, respectively; and most are perennial, 100% and 90%, respectively. The WI plants typically had a narrower native distribution than OI, but species of both types present a multicontinental distribution almost as frequently. The increase in the multicontinental distribution from 6% to 63% in WI reflects their strong capacity for global dispersal and establishment. High proportions of both WI and OI possess a mixed reproduction system, i.e., both sexual and asexual modes of reproduction, equaling 57% and 50%, respectively, which brings flexibility to their spreading and colonization. Sexual reproduction is less common in WI species, and when reproducing sexually they have a stronger tendency towards hermaphroditism. In particular, hermaphroditism combined with self-compatibility may be a factor that enhances invasiveness, allowing fertilization and recombination even under low population densities.

## Figures and Tables

**Table 1:** Characteristics of invasive plants: 35 plant species belonging to the 100 world’s worst invasive alien species of any kind vs. 432 other seriously invasive alien plant species.

Trait	Worst Invasive	Other Invasive
Dispersal and Distribution		
Strongly human-assisted dispersal	30 (86%)	392 (91%)
Multicontinental original distribution	2 (6%)	93 (22%)
Multicontinental present distribution	22 (63%)	253 (59%)
Typically in disturbed habitats (excl. aquatic taxa)	30 (94%)	325 (80%)
Uses		
Mainly ornamental	21 (60%)	229 (53%)
Other known uses	8 (23%)	103 (24%)
No known use	6 (17%)	100 (23%)
Life History and Reproduction		
Perennial	35 (100%)	388 (90%)
Mainly/only sexual	8 (23%)	171 (40%)
Mainly/only asexual	7 (20%)	44 (10%)
Commonly sexual and asexual	20 (57%)	217 (50%)
Distribution of Sexual Function (incl. angiosperms)		
Hermaphroditic (excl. monoecious)	25 (80%)	263 (64%)
Dioecious	6 (20%)	82 (20%)
Monoecious	0 (0%)	66 (16%)

## Data Availability

All data generated or analyzed during this study are included in this article.

## Supplementary Materials

The following is available online at https://www.mdpi.com/article/10.3390/life13071596/s1, Table S1: Basic background information of plant species considered invasive based on the Invasive Species Specialist Group of IUCN (www.issg.org). The most harmful invasive plant species are marked with a red background color.
